# Interferon-β 1a and SARS Coronavirus Replication

**DOI:** 10.3201/eid1002.030482

**Published:** 2004-02

**Authors:** Lisa E. Hensley, Elizabeth A. Fritz, Peter B. Jahrling, Christopher Karp, John W. Huggins, Thomas W. Geisbert

**Affiliations:** * U.S. Army Medical Research Institute of Infectious Diseases, Fort Detrick, Maryland, USA; † Cincinnati Children’s Hospital Medical Center, Cincinnati, Ohio, USA

**Keywords:** SARS, interferon, coronavirus, treatment, therapy

## Abstract

A global outbreak of severe acute respiratory syndrome (SARS) caused by a novel coronavirus began in March 2003. The rapid emergence of SARS and the substantial illness and death it caused have made it a critical public health issue. Because no effective treatments are available, an intensive effort is under way to identify and test promising antiviral drugs. Here, we report that recombinant human interferon (IFN)-β 1a potently inhibits SARS coronavirus replication in vitro.

The recent global outbreak of severe acute respiratory syndrome (SARS) has quickly gained notoriety as a newly emerging infectious disease. The etiologic agent was identified as a coronavirus (SARS-CoV) that is not closely related to any of the previously characterized coronaviruses (*1**,**2*). As of September 26, 2003, a total of 8,098 probable cases of SARS have occurred with 774 deaths.

No antiviral treatments are currently available against SARS-CoV. SARS cases have been treated symptomatically according to the severity of the illness. A treatment protocol consisting of antibacterial agents and a combination of ribavirin and methylprednisolone was recently proposed. However, the therapeutic value of ribavirin remains uncertain because it has no activity against SARS-CoV in vitro. Molecular modeling studies suggest that rhinovirus 3C^pro^ inhibitors may be useful for SARS therapy, but results of recent in vitro testing of the lead molecule, AG7088, were negative (*3*).

Previous studies showed that some coronaviruses, including avian infectious bronchitis virus, murine hepatitis virus, and human coronavirus 229E, are susceptible to type I interferons in vitro or in vivo (*4**–**7*). Therefore, we evaluated the in vitro efficacy of a recombinant human type I interferon (IFN), IFN-β 1a (Serono International, Geneva, Switzerland) against three different isolates of SARS-CoV (Tor2 and Tor7 and Urbani) using yield reduction assays. The IFN-β 1a preparation employed in this study was selected because it is currently used as part of the most effective treatment regimen for relapsing forms of multiple sclerosis (*8*), and more importantly, because it was shown to have antiviral activity (as measured in a vesicular stomatitis virus cytopathic assay system) 14 times greater than the currently available treatment using IFN-β 1b (*9*).

In the current study, Vero E-6 cells were treated with concentrations (5,000 to 500,000 IU/mL) of IFN-β 1a either 24 h before or 1 h after inoculation with the SARS-CoV (m.o.i. 0.1 PFU/cell), and monitored for cytopathic effect and production of infectious SARS-CoV at 24, 48, and 72 h postinfection. Inhibition of the SARS-CoVs by IFN-β 1a was dependent on both time of drug administration and time of culture sampling after SARS-CoV infection. Production of infectious SARS-CoV was potently inhibited (≥99.5% or 2.00 log10 PFU/mL) at 24 h postinfection. by pretreatment of Vero E-6 cells with IFN-β 1a at all concentrations tested (Figure 1). By 72 h postinfection, inhibition of SARS-CoV production by IFN-β 1a had declined for all three SARS-CoVs, with inhibition (>70%) being detected in the Tor7 (Figure 1) and Urbani isolates (data not shown). IFN-β 1a was somewhat less effective at inhibiting SARS-CoV replication when employed after infection of cultures (Figure 1). Nonetheless, production of infectious SARS-CoVs was considerably reduced (≥90% or 1.00 log10 PFU/mL) at 24 and 48 h postinfection. Protection of Vero E-6 monolayers against SARS-CoV–induced cytopathic effects by preinfection or postinfection treatment with IFN-β 1a was dramatic, even at 72 h postinfection (Figure 2). Additional concentrations of IFN-β 1a (0.5–5,000 IU/mL) were tested to determine the 50% inhibitory concentration (IC_50)_. Pretreatment of Vero E-6 cells with concentrations as low as 50 IU/mL, or posttreatment of cells with concentrations at 500 IU/mL, provided a 50% reduction with the Tor2 isolate at 24 h postinfection.

**Figure 1:**
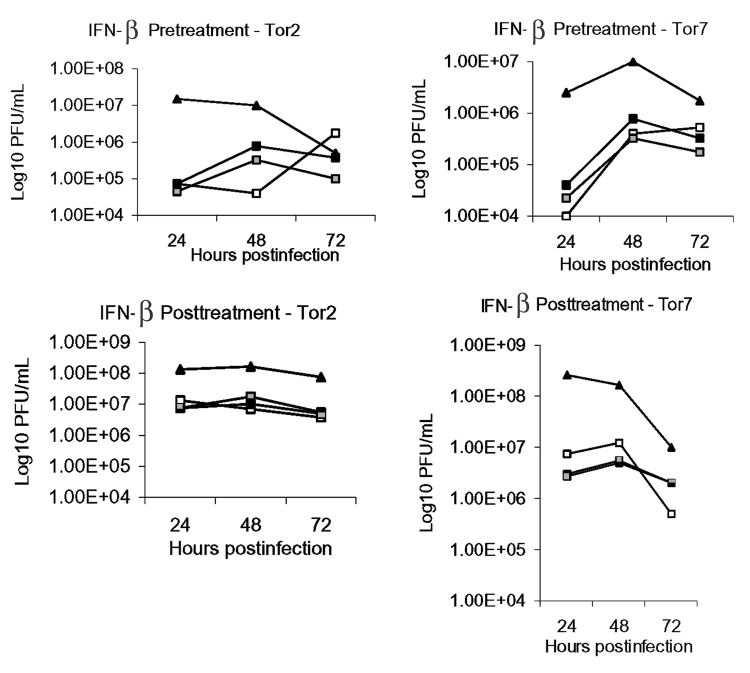
Interferon (IFN)-β 1a inhibition of SARS-CoV replication in Vero E-6 cells. Top panels, Vero E-6 cells were incubated in the absence (-▲-) or presence of IFN-β 1a added 24 h before infection with the Tor2 (left) or Tor7 (right) isolate of SARS Co-V. Bottom panels, Vero E-6 cells were incubated in the absence (-▲-) or presence of IFN-β 1a added 1 h after infection with the Tor2 (left) or Tor7 (right) isolate of SARS Co-V. Three concentrations of IFN-β 1a were employed for both studies: 5,000 IU/mL (-□-), 50,000 IU/mL (-■-), 500,000 IU/mL (-■-) Samples of overlying media were collected at 24, 48, and 72 h postinfection and analyzed by plaque assay on Vero E-6 cells.

**Figure 2:**
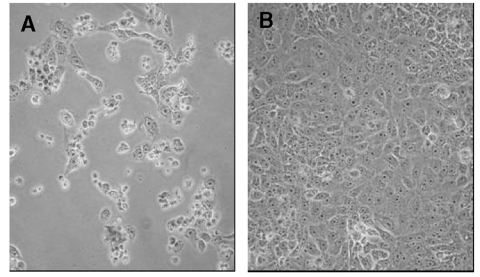
Interferon (IFN)-β 1a inhibition of SARS-CoV cytopathicity in Vero E-6 cells. Vero E-6 cells were infected with the Tor2 isolate of SARS-CoV and incubated for 72 h in the absence (left panel) or presence (right panel) of 500,000 IU of recombinant human IFN-β 1a. Cell rounding and detachment were prominent in the absence of IFN-β 1a. Minimal cell rounding or death was noted in the intact monolayer at 72 h postinoculation in the presence of IFN-β 1a (note: IFN-β 1a administered 1 h postinfection).

Faced with a burgeoning epidemic of SARS cases and a lack of effective treatment options, identifying compounds with antiviral activity that could be potential therapeutics has become a high priority. Our report suggests that IFN-β 1a may be effective as a treatment for SARS-CoV infections. As noted above, IFN-β 1a is currently being used for a variety of clinical indications, including multiple sclerosis, and has shown dose-dependent efficacy in several clinical trials. Importantly, IFN-β 1a exhibited potent antiviral activity at doses that have already been shown to have acceptable safety profiles in animals (*10*). Thus, we report the identification of a compound that may be suitable for rapid development as a treatment for SARS-CoV infection.
